# A Multivariate Surface-Based Analysis of the Putamen in Premature Newborns: Regional Differences within the Ventral Striatum

**DOI:** 10.1371/journal.pone.0066736

**Published:** 2013-07-03

**Authors:** Jie Shi, Yalin Wang, Rafael Ceschin, Xing An, Yi Lao, Douglas Vanderbilt, Marvin D. Nelson, Paul M. Thompson, Ashok Panigrahy, Natasha Leporé

**Affiliations:** 1 School of Computing, Informatics, Decision Systems and Engineering, Arizona State University, Tempe, Arizona, United States of America; 2 Department of Radiology, Children’s Hospital of Pittsburgh, University of Pittsburgh Medical Center, Pittsburgh, Pennsylvania, United States of America; 3 Department of Radiology, Children’s Hospital Los Angeles, Los Angeles, California, United States of America; 4 Department of Biomedical Engineering, University of Southern California, Los Angeles, California, United States of America; 5 Department of Pediatrics, University of Southern California, Los Angeles, California, United States of America; 6 Developmental-Behavioral Pediatrics Fellowship Program, Children’s Hospital Los Angeles, Los Angeles, California, United States of America; 7 Department of Radiology, University of Southern California, Los Angeles, California, United States of America; 8 Imaging Genetics Center, Laboratory of Neuro Imaging, University of California Los Angeles School of Medicine, Los Angeles, California, United States of America; Institute of Psychology, Chinese Academy of Sciences, China

## Abstract

Many children born preterm exhibit frontal executive dysfunction, behavioral problems including attentional deficit/hyperactivity disorder and attention related learning disabilities. Anomalies in regional specificity of cortico-striato-thalamo-cortical circuits may underlie deficits in these disorders. Nonspecific volumetric deficits of striatal structures have been documented in these subjects, but little is known about surface deformation in these structures. For the first time, here we found regional surface morphological differences in the preterm neonatal ventral striatum. We performed regional group comparisons of the surface anatomy of the striatum (putamen and globus pallidus) between 17 preterm and 19 term-born neonates at term-equivalent age. We reconstructed striatal surfaces from manually segmented brain magnetic resonance images and analyzed them using our in-house conformal mapping program. All surfaces were registered to a template with a new surface fluid registration method. Vertex-based statistical comparisons between the two groups were performed via four methods: univariate and multivariate tensor-based morphometry, the commonly used medial axis distance, and a combination of the last two statistics. We found statistically significant differences in regional morphology between the two groups that are consistent across statistics, but more extensive for multivariate measures. Differences were localized to the ventral aspect of the striatum. In particular, we found abnormalities in the preterm anterior/inferior putamen, which is interconnected with the medial orbital/prefrontal cortex and the midline thalamic nuclei including the medial dorsal nucleus and pulvinar. These findings support the hypothesis that the ventral striatum is vulnerable, within the cortico-stiato-thalamo-cortical neural circuitry, which may underlie the risk for long-term development of frontal executive dysfunction, attention deficit hyperactivity disorder and attention-related learning disabilities in preterm neonates.

## Introduction

Frontal executive dysfunction (FED) and behavioral problems including attentional deficit/hyperactivity disorder (ADHD) have been observed over the long term in survivors of prematurity [1]. Abnormalities in cortico-striato-thalamocortical circuits may underly defects in both FED [2] and ADHD [1]. Most of the recent research on neural substrate deficiencies that underlie neurocognitive problems in preterm subjects has focused on thalamo-cortical pathways, with very little focus on the putamen (see e.g. [3], [4]), or other structures of the striatum.

Nonspecific volumetric deficits of striatal structures have been found in preterm neonates and children [5]–[7]. For example, a whole-brain deformation-based morphometry study [8] found significant reduction of the lentiform nuclei in premature neonates compared to term-born controls. In addition, using voxel-based morphometry, Nosarti and colleagues [9] detected differences in the putamen in adolescents born very prematurely. One volumetric study in preterm neonates also found altered volumes in a segmentation containing both the thalamus and basal ganglia, but did not perform individual regional assessment of these structures [10].

These findings highlight the need to assess the putamen in more detail in developing preterm neonates. However, none of the morphometric studies in these subjects to date has focused specifically on the striatal structures including the putamen. In addition, all studies so far have been whole-brain volumetric ones, but as shown in several prior works (see e.g. [4], [11], [12]) important complementary information may be found through surface-based analyses. Abnormalities of the putamen have been implicated in impaired visual and motor functions, and learning disabilities in preterm subjects [13]. Determining morphological differences in this structure can help in understanding how grey matter abnormalities correlate with outcome in preterm neonates.

**Figure 1:**
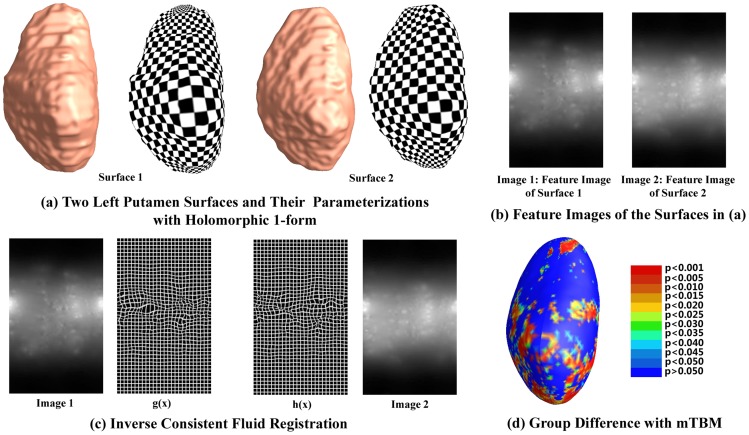
The proposed system. (a) The putamen is segmented from T1-weighted images and a conformal grid is built on the surface. Here examples are shown for 2 different subjects (b) Examples of features selected in the image for the two subjects in (a) (c) From left to right: Intensity map on the surface of one of the subjects in (a). Forward map g(x) for the conformal grid fluid registration to a common template. Backward map h(x) from the template to image 1. Intensity map on the surface of the template. (d). Surface mTBM is applied to analyze morphometric changes. Map displays uncorrected p-values on the surface of the putamen from the mTBM analysis.

Here, we perform the first ever surface morphometry analysis of the putamen in premature neonates. We tested the hypothesis that there is regional vulnerability of the striatum within the frontal-striatal neural circuitry of preterm neonates. More specifically, starting from a volumetric dataset of T1-weighted brain MRI, we compared the manually segmented striatal morphology of 17 preterm and 19 term-born newborn subjects using a pipeline that we recently implemented for the surface analysis of subcortical structures in neonates (Fig. 1; see [4], [11], [12]). We aimed to detect surface-based regional shape differences between the two groups. Our method involves conformal grid generation on the surface [14], surface fluid registration [15] and a multivariate tensor-based morphometry statistical analysis [17]; this type of analyses has been shown to increase detection power in our neonatal studies [4], [12], and in adults [17]. We analyzed preterm neonates with no visible evidence of white matter injury, to determine the effects of prematurity on regional surface deformation measures without the confounding factor of white matter injury. Our work presents the first precise subparcellation of the putamen in premature neonates and provides more regional specificity than prior group analyses of these subjects.

## Materials and Methods

### Neonatal Data

Our dataset comprises 17 premature neonates (gestational ages 25–36 weeks, 41.1 ± 5.0 weeks at scan time) with normal MR scans and 19 healthy term born infants (45.1 ± 5.1 weeks at scan time).

MRI scans were acquired using a dedicated neonatal head coil on a 1.5T GE scanner and include a coronal 3D SPGR sequence (TE = 6 ms; TR = 25 ms, FOV = 18 cm; matrix = 256×160), axial and sagittal T1-weighted FLAIR sequences (TE = 7.4, TR = 2100; TI = 750; FOV = 20 cm; Matrix = 256×160) and an axial T2-weighted FSE sequence (TE = 85 ms, TR = 5000 ms, FOV = 20 cm, matrix = 320×160 or 256×128).

Inclusion criteria for our preterm subjects were the following: 1) prematurity, and 2) visually normal scans on conventional MRI. Structural MRI were qualitatively classified as controls by 2 board-certified neonatal neuroradiologists.

This study was approved by the CHLA Committee on Clinical Investigations and the University of Pittsburgh Internal Review Board. Written consents for use of their child’s clinically acquired MRI data and for participation in additional neurodevelopmental and neuroimaging studies were obtained from parents on behalf of the prospectively recruited patients by a research coordinator. The ethics committee approved this consent process. Additionally, as this study involved a retrospective review of all clinically-acquired neonatal data for the period between 2005 and 2011, which included neonates who were not enrolled into prospective studies, approval has also been obtained from the CHLA Committee on Clinical Investigations and the University of Pittsburgh Internal Review Board for the retrospective use of all clinically-acquired neonatal MRI data acquired at CHLA between 2005 and 2011.

**Figure 2:**
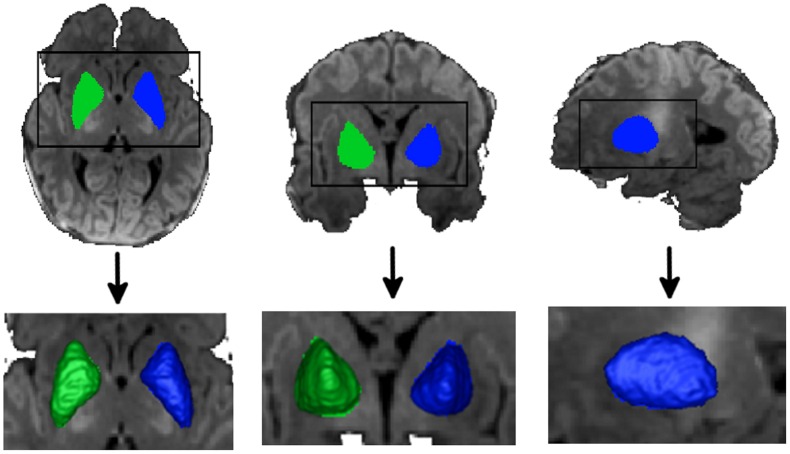
Putamen segmentation. While the caudate head is part of the striatum, we chose not to include this structure because of the poor tissue contrast in the anterior limb of the internal capsule in the neonates, which prevented us from obtaining reliable manual contours in this region in neonates. Additionally, our hypothesis testing for this paper included the ventral striatum, which does not contain the caudate head. Note also that the globes palladius, while not part of the putamen, was still included in our segmentation. It is extremely difficult to accurately segment the globus pallidus from the putamen in neonates due to the lack of tissue contrast on volumetric T1 images. However, since our hypothesis is related to the ventral striatal surface, it is not affected by the inclusion of the globus palladius within our putamen contour.

We manually segmented the putamen with Insight Toolkit’s SNAP program [18], as shown in Fig. 2. Tracings were performed in the registered template space by an experienced pediatric neuroradiologist, using standard protocols. We consulted neuranatomical references of the human striatum to help guide the placement of the contours [19], [20]. Our segmentation of the putamen did not include the caudate head or the globus pallidus, and was defined laterally by the insular cortex/claustrum/external capsule margin and medially by the anterior limb of the internal capsule and the genu and posterior limb of the internal capsule. We did likely include the nucleus accumbens within the inferior and medial aspect of the contour as this structure is known to be difficult to separate, on MRI, from the adjacent putamen and the basal forebrain region. Our inter-rater average percent volume overlap (intersection of volumes over average of them) for 4 structures segmented twice at a few months interval is 86%.

Figs. 1 a) shows examples of reconstructed putamen surfaces.

### Surface Conformal Grid Generation and Registration

We computed conformal grids on the putamen using holomorphic differentials as described in [14]. Our grid generation method starts with the assumption that some geometrically extreme positions can serve as geometrically valid and consistent landmarks across subjects on the putamen. Hence, we introduce two cuts at the extremities of the putamen. The putamen is consistently bordered by the external capsule laterally, the anterior limb of the internal capsule anteriorly and the posterior limb of the internal capsule posteriorly. We call this step a topology optimization, as it introduces consistent landmarks via a topology change. These landmarks are used to achieve an accurate surface registration (see below), a prerequisite of tensor-based morphometry analyses. This approach was successfully used in several of our prior work with different subcortical structures such as the hippocampus [16], lateral ventricle [17], thalamus [4] and corpus collosum [11].

After the topology optimization is performed, the putamen surface is represented as a genus-zero surface with two open boundaries, i.e. a topological cylinder. We compute its conformal parameterization with a holomorphic 1-form method [14]. Briefly, by solving Laplace equations on the surface, we compute a harmonic 1-form and its conjugate harmonic 1-form and, following Hodge Theorem, we generate the holomorphic 1-form by pairing them. By integrating this holomorphic one-form, a conformal mapping is found from the putamen to a rectangle and introduce a conformal grid on the putamen surface. Examples of grids that we generated are shown in Fig. 1(a).

We then map the surface grids to a common template (i.e., to one of the controls, chosen at random) with a new fluid registration technique that we recently implemented [15]. We use surface features composed of the surface conformal factor and mean curvature to enforce surface correspondence. Similar to prior research [21], [22], a flow computed in the parameter space (a planar domain in our case) of the two surfaces, induces a correspondence field in 3D. To simulate fluid flow on the surfaces, the Navier-Stokes equation is extended into surface space using a manifold version of the Laplacian and divergence operators [23], [24]. With an inverse consistent framework, surface registration is optimized when the sum of squared surface feature intensity differences between the deforming image and template image is minimized [15]. Fig. 1 (b) shows the computed surface feature images and (c) illustrate the inverse consistent fluid registration process on the parameter space.

Since both the conformal mapping and inverse consistent fluid registration generate diffeomorphic mappings, the mapping between the putamen surfaces is diffeomorphic.

To confirm the validity of our analysis, we also compared the fluid registration method to the more usual constrained harmonic map registration [16]. Given two surfaces 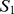 and 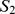, the latter consists of finding a harmonic map between their parameter domains (here a planar surface). Surface matching is achieved by composing the conformal map from 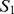 to its parameter domain 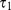, the harmonic map between 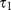 and 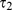 (the parameter domain of 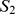), and the inverse conformal map between 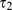 and 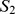.

Both the constrained harmonic map and the inverse consistent fluid registration methods are valid approaches to subcortical surface registration. In particular, the latter method extends the standard volume-based 3D fluid image registration approach to surfaces by generating a diffeomorphic mapping that minimizes surface features differences. In other words, the surface fluid registration method generates a mapping that enforces key surface features alignment. As a result, we hypothesized here that it would be consistent with but outperform the constrained harmonic mapping by generating more accurate and biologically meaningful surface correspondences.

### Surface Multivariate Tensor-based Morphometry

Our ultimate aim is to determine the intrinsic surface morphology of segmented putamens in preterm neonates. We do so by applying a multivariate tensor-based morphometry analysis [16], [17], [25].

In tensor-based morphometry, for each subject in the data set, the registration yields a displacement field 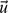 between the template and the subject’s images. A Jacobian matrix 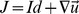 is computed at each vertex from the registration between template and subjects images, where 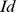 is the identity matrix. These Jacobian matrices, or a function of their components are used as metrics for group comparisons. For example, the determinant, 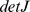 expresses the ratio of the surface area between the moving and fixed images while in multivariate tensor-based morphometry, we use the deformation tensors 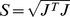 (in fact their logarithm [17], [25], [26]). 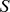 can be represented as a 2D ellipse at the center of each grid cell, whose axes show the direction and size of the change in area between the two surfaces at that location. In general, the multivariate measures yield increased statistical power when compared to the univariate ones (see e.g. [25] for the volume-based multivariate tensor-based morphometry and [4], [17] for the surface-based one).

### Medial Axis Method

One of the most commonly used morphometry measure on surface data is the radial distance 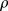 from a medial axis to a vertex of the surface [27], [28]. We used the medial axis method as a complementary measure to the surface shape analysis from multivariate tensor-based morphometry. Here the medial axis was computed using the center point of the iso-parametric curves, on the conformal grid [16].

### Multivariate Statistical Analysis

We used a total of four different statistics: the deformation tensors 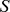, the radial distance 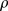, the determinant of the Jacobian matrix 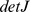 and a vector 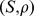 containing the elements of 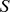 and 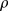.

To adjust for post-conception age effects, we first covaried the measures at each vertex with this variable. Let A represent one of the four statistic, and Acov is the new adjusted statistic. The Acov were computed by fitting the following general linear model to the data at each image voxel:
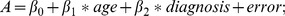
where the 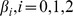 are estimated regression coefficients at that specific vertex. Diagnosis was coded as a binary dummy variable (that is, diagnosis = 0 (term-born) and 1 (preterm)), so that 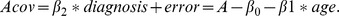 For the multivariate measures, the regression was computed separately for each channel.

For 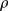 and 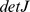, a standard voxel-wise 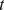-test was used to compare the preterm to the term-born neonates, while for either of the multivariate tests on 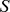 or 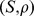, group statistics were computed using the Hotelling’s 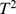 test [29] - the multivariate extension of the Student’s 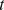-test -, as described in [16], [17], [25].

In order not to assume a normal distribution, we ran a vertex-based permutation test on the results [17], [25], [30]. We randomly assigned diagnostic labels (premature or term-born) to each subject, without replacement. A 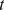
- or 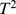-test was performed at each vertex using the new labels. The procedure was repeated 10000 times. We obtained a null distribution of 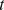
- or 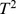-values at each vertex, to which we compared the 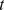
- or 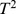-values from the real data. Fig. 1 (d) shows an example 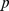-map in a group difference study where mTBM are adopted as morphometry features.

We also performed a second permutation test for multiple comparison corrections. We again randomly shuffled labels between the two groups, but this time we computed a single value on each image, that is, the number 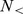 of vertices with 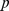-values 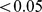. This provided us with a null distribution to which we compared 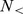 from the real data.

We also created cumulative distribution function (CDF) plots of the resulting uncorrected 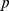-values (as in a conventional false discovery rate analysis (FDR) [31]). The critical 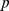-value [31] is the highest non-zero point at which the CDF plot intersects the 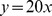 line, and represents the highest statistical threshold (if there is one) that can be applied to the data, for which at most 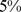 false positives are expected in the map. If there is no such intersection point (other than the origin), there is no evidence to reject the null hypothesis.

## Results

**Figure 3:**
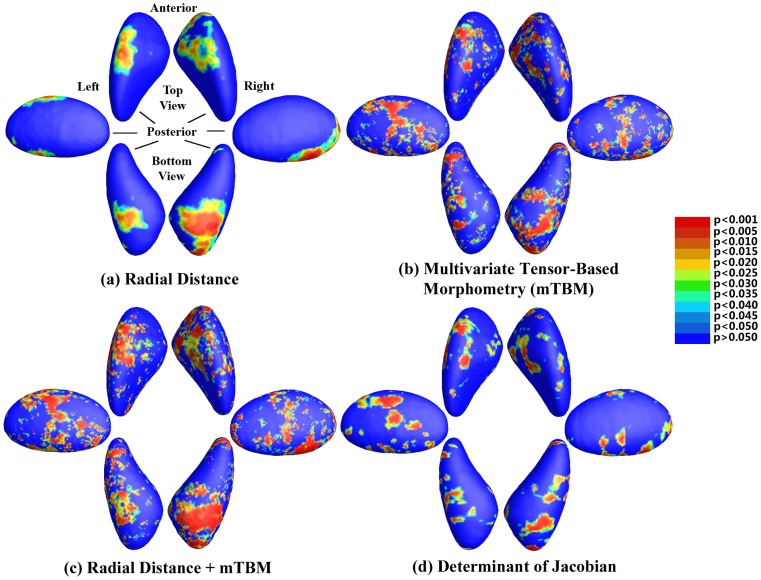
Surface-based statistics. Top panel: P-values for the comparison of two groups, for 4 different statistics. The meaning of the different colors is shown in the colorbar.

Figure 3 shows the surface maps for all four statistics. Clusters of significance are found bilaterally, particularly on the anterior and inferior surface of the putamen. All measures give similar clusters of significance, though the multivariate measures are more powerful (but noisier) than the univariate ones. While all measured reached significance using a threshold of 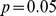, the combined one 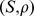 had the lowest 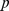-value. Clusters are primarily found in the anterior/inferior surface of the putamen, bilaterally. Of note, there are relatively less significant clusters seen in the region of the lateral, superior and medial surface of the striatal structure, and in the region of the globus pallidus. The caudate head, which is also considered a striatal structure, was not included within the manually delineated contour.

**Figure 4:**
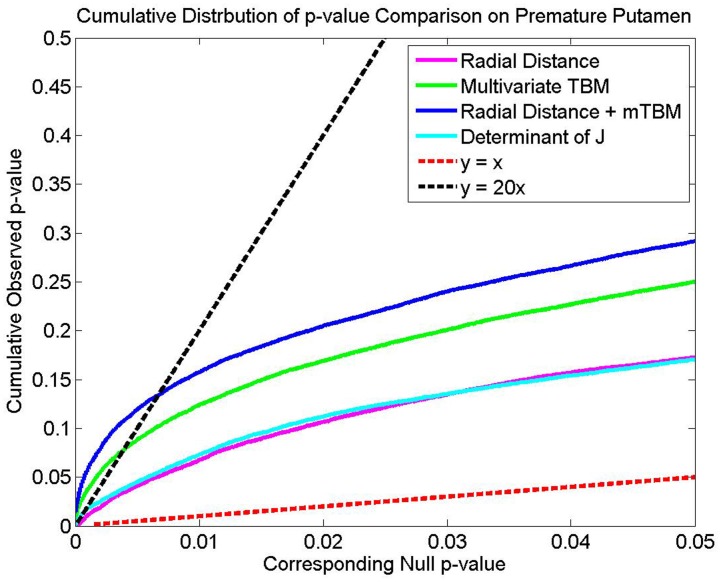
False Discovery Rate Analysis. This plot shows the CDFs of p-values vs. p-values. Height above the x = y line indicate how powerful a statistic is, while the point at which the data crosses the y = 20× line, if it exists, represents the threshold at which at most 5% of false positives are expected (see section on Multivariate Statistical Analysis). Both mTBM and the combined measure cross the x = 20 y line but the other measures fail to do so.

Fig. 4 shows the result of the FDR analysis, for which the CDFs of the 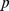-values are plotted against the 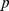-values. Neither of the univariate measures cross the 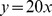 line, and hence, there is no evidence to reject the null hypothesis for these measures. However, both the mTBM and the combined mTBM and radial distance fall above this threshold, with the combined measure outperforming mTBM alone.

These results are further confirmed by our whole map 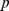-value analysis. Corrected 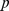-values for multiple comparison using the permutation method were: (a) 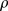: 0.045; (b) 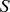: 0.0016; (c) 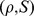: 0.0011; (d) 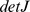: 0.029. In this case, the univariate measures are marginally significant given a significance threshold of 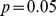, but are strongly outperformed by both mTBM and the combined measure.

**Figure 5:**
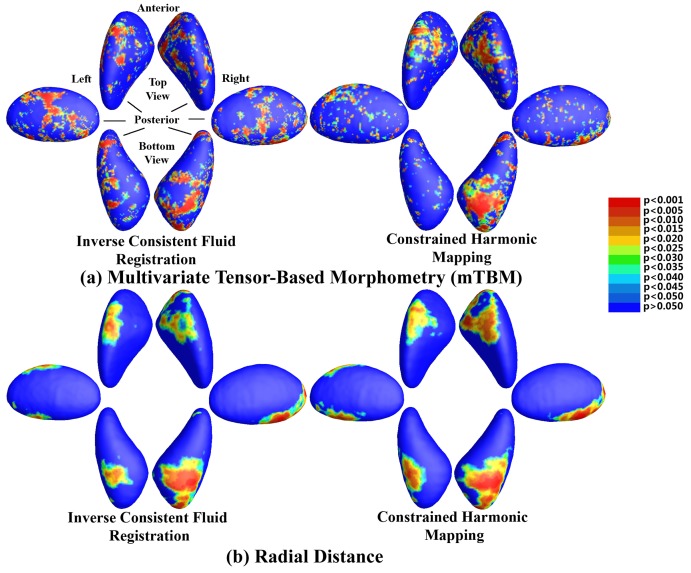
Comparison of Fluid and constrained harmonic registrations. Top panel: P-values for the comparison of 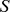 with the two registration methods. Bottom panel: P-values for the comparison of 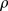 with the two methods. The left column represents the fluid registration, while the right one is for the constrained harmonic mapping. The meaning of the different colors is shown in the colorbar.

Fig. 5 shows a comparison of the fluid registration method compared to the constrained harmonic one. The two methods give similar clusters of significance, which provides a validation of the fluid registration method. Corrected 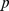-values for multiple comparison using the permutation method for the constrained harmonic maps were: (a) 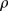: 0.089; (b) 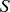: 0.055; (c) 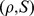: 0.036; (d) 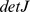: 0.097. The fluid registration results are noiser for surface-based statistics, though not for the radial ones, but they give results that are more powerful.

## Discussion

We applied a novel pipeline for surface based analysis of subcortical structures of neonates to compare the putamen in premature neonates to term born controls. We detected widespread areas of significance throughout the putamen, with focal and regional involvement of the anterior and inferior surface of the putamen within the ventral striatum. All measures gave consistent results, but statistics on the deformation tensors outperformed both univariate measures, and the combined 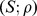 statistic also outperformed S alone. Both 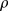 and 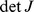 gave similar clusters of significance, though the former gives radial information, while the latter describes changes constrained to the surface.

In addition, we compared the surface fluid registration to the constrained harmonic map, and the former outperformed the latter in terms of statistical significance.

### 

#### Methodological Considerations

Our premature and control groups were slighly mismatched in age at scan time. While we covaried for this variable in the analysis, non-linear age effects will not be removed from the linear regression analysis. In a recent study of the thalamus, we found that the effect of age at scan time on the size of the thalamus was roughly linear on that structure [Lao et al., Medical Image Computing and Computer Assisted Intervention, submitted, 2013], and a similar result was found for the putamen [Lao et al., in preparation]. This does not preclude small non-linear differences in other local parameters such as regional surface areas, and we recognize this as a caveat of our study. We plan on studying these non-linear effects in future studies with larger data sets.

As expected, the multivariate surface analysis (mTBM) outperformed the univariate determinant of the Jacobian matrix (TBM). This result is in line with several other comparison studies comparing mTBM to TBM, both on subcortical surfaces [4], [17], and on the whole brain volume [25]. This result is not trivial: while adding additional information may increase statistical power, some channels may be noisier and contribute to reducing the overall signal. Additionally, radial distance results may or may not correspond to those on the mTBM ones, as they measure orthogonal and hence complementary quantities. In this work, we find similar clusters of changes between the two measures, though p-values are lower for areal changes. These results demonstrate the usefulness of adding surface area analyses to the more traditional radial ones.

The surface fluid registration and the constrained harmonic mapping are similar in nature as each of them converts the surface matching problem to a familiar 2D image registration via a conformal parametrization. The harmonic method relies more on geometry, and, on a convex polygon, the harmonic map is always a diffeomorphism. The limitation here is that results strongly depend on the Dirichlet boundary conditions. On the other hand, the surface fluid method allows for large diffeomorphisms, and improves the registration by considering the important shape or functional features as image features for registration, so that it is more general and reduces the dependence on the boundary conditions. However, its results are not in a very tight connection to surface intrinsic geometric features as we model shape features as general explicit constraints.

#### Neuroanatomic Considerations

Our findings suggest that there is regional involvement of the anterior inferior putamen within the ventral striatum in preterm neonates. How is this relevant to understanding the cortico-striatal-thalamo-cortical circuitry in preterm subjects?

There is controversy regarding the functional and neuroanatomic subparcellation of the striatum, which has mostly been determined using animal models [32]. However, recently, Draganski et al. studied the in vivo human spatial extent and topography of basal ganglia connectivity using probabilistic tractography techniques [33]. Their study showed that the most anterior and inferior aspect of the putamen in adults appears to be interconnected with the medial orbital and pre-frontal cortex. The same region of the anterior and inferior aspect of the putamen is also interconnected with the midline nuclei of the thalamus including the medial dorsal nucleus and the pulvinar. Medial-orbital/pre-frontal cortex, medial-dorsal and pulvinar abnormalities have been detected in preterm subjects in prior neuroimaging studies [3], [34]–[36]. Our results add to this body of literature and suggest that a specific neural network within the cortico-striatal-thalamo-cortical circuitry is at risk in preterm subjects. To further validate this hypothesis, future work is needed, correlating our putamen findings with cortical and thalamic segmentations and cortico-thalamic probabilistic tractography in preterm subjects.

Others have shown that frontal-striatal pathways are abnormal in older preterm subjects using functional MRI [37]. Frontal cortical regions - such as the prefrontal and medial orbital areas - are interconnected with the anterior and inferior putamen, but there are also limbic connections with the nucleus accumbens of the ventral striatum. The lateral aspect of the nucleus accumbens may overlap with some of the significant clusters of the anterior and inferior surface of the putamen. Even so, the nucleus accumbens is difficult to distinguish on volumetric neonatal T1- and T2-weighted MRI. The nucleus accumbens is a critical structure in the mesocortical-limbic circuitry [38] and there is evidence that the limbic system is abnormal in preterm subjects.

#### Neuropathological Considerations

What may be the underlying pathological substrate of regional differences in the anterior and inferior surface of the putamen in our study? In our study, we excluded preterm infants with visible evidence of either grey matter or white matter injury on conventional MRI. There may have been subtle direct injury to the putamen, which has been demonstrated in other preterm studies [39]. It is also possible that there might be mild diffuse white matter injury (undetectable on conventional MRI) in our preterm cohort. This may indirectly alter the cortico-striatal-thalamo-cortical neural circuitry described above. Depending on the degree of involvement of the nucleus accumbens, which is adjacent to the anterior and inferior margins of the putamen, both subtle grey and white matter injury may have also altered the mesocortical-limbic pathways in our preterm subjects. This constellation of both grey and white matter abnormalities is part of the encephalopathy of prematurity as proposed by Volpe et al [40]. Regional differences seen in the putamen in our study may relate to experience dependent changes or environmental/exogenous stressors on morphology and neural circuitry in our preterm subjects, but more validation would be needed to prove this.

#### Clinical Application

As neonatal clinical care has improved, survival rates have increased for preterm low birth weight children, but they are still at risk for developmental problems. Major developmental disabilities (including cerebral palsy) can be detected in early infancy, but these are not so prevalent. In contrast, the incidence of lower-severity disabilities including ADHD, FED and specific learning disabilities has increased in preterm children, and are detected later at school age. Techniques are needed that can detect specific neural pathways at risk for attention deficits in preterm subjects. Infants and preschool children who are preterm show less efficient attention behaviors than their term born counterparts [41]. These attentional deficits in preterm subjects likely underlie the risk for specific learning disabilities, FED and ADHD later in life. Understanding the neural substrates of attentional problems in preterm infants, including the influences of biological and environmental factors, is critical for developing effective interventions.

An important clinical implication of accurate putamen morphometry pertains to the hypothesis that striatum differences among premature neonates may account for their high rates of ADHD [1]. In fact, a recent meta-analysis found reduced right globus pallidus and putamen volumes in children with ADHD [42]. If these putamen characteristics can identify early ADHD, then behavioral or psychopharmacological treatments may be brought to bear in minimizing problematic social, behavioral, emotional and learning outcomes. Cross-sectional studies of stimulant treated ADHD patients have shown reduced volume and voxel-based morphometric abnormalities in these subjects compared to non-treated ADHD groups [42], [43]. If these findings can be confirmed, this suggests possible normalization of striatal structure with appropriate pharmacological treatment.

Closely aligned with ADHD is executive functioning, which allows a child to selectively inhibit responses and switch tasks while performing cognitive operations. Significant relationships exist between spatial working memory, which is a measure of executive function, and volumetric measurements of the putamen [44]. Early identification of morphological changes that may be associated with executive functioning deficits would allow learning based interventions.

Understanding the link between putamen morphology and limbic system function is also clinically relevant based on the high co-morbidity of ADHD with depression and anxiety, especially in adolescents [45]. In this study, Biederman et al. found that teenagers with ADHD showed surface abnormalities in the ventral or anterior putamen, similar to our results. This suggests that in these children, cortical-striatal-cortical thalamic connection differences may set up a vulnerability for ADHD. As the anterior putamen is interconnected to the limbic system, preterm neonates who may have subtle brain injury may be at heightened risk of developing ADHD and learning deficits [32].

#### Future Directions

We are currently working on increasing the sample size to confirm the above results. In larger studies in adults, multivariate tensor-based morphometry outperformed univariate methods in detecting brain differences in lateral ventricles in HIV/AIDS patients [17], and in a large Alzheimer’s morphometry study (804 subjects) of subcortical structures [16]. We expect that this will be the case in neonates too.

In the future, we aim to correlate the above results with clinical outcomes, and we will also perform group comparisons of the putamen between older premature and control children to see if the differences found here disappear, or are reduced, with age. The methods described here could be used for early detection or risk stratification of neurodevelopmental problems such as ADHD, internalizing problems and executive function and other learning deficits so that innovative therapies or early interventions could be administered. In the immediate neonatal period, our morphometric techniques could also be used to determine the effect of certain therapies (i.e., antibiotics, hypothermia, and/or pharmacologic agents) on the development of the putamen. We have applied the methods here to preterm neonates, but the methods described above may be applied to study group differences in this structure due to various neurological conditions in subjects of all ages. Our pipeline may be downloaded freely http://gsl.lab.asu.edu/conformal.htmon our website.
